# Thymus Degeneration and Regeneration

**DOI:** 10.3389/fimmu.2021.706244

**Published:** 2021-09-01

**Authors:** Maxwell Duah, Lingling Li, Jingyi Shen, Qiu Lan, Bin Pan, Kailin Xu

**Affiliations:** ^1^Department of Hematology, The Affiliated Hospital of Xuzhou Medical University, Xuzhou Medical University, Xuzhou, China; ^2^Blood Diseases Institute, Xuzhou Medical University, Xuzhou, China

**Keywords:** thymus, thymic epithelial cells, degeneration, regeneration, autoimmune

## Abstract

The immune system’s ability to resist the invasion of foreign pathogens and the tolerance to self-antigens are primarily centered on the efficient functions of the various subsets of T lymphocytes. As the primary organ of thymopoiesis, the thymus performs a crucial role in generating a self-tolerant but diverse repertoire of T cell receptors and peripheral T cell pool, with the capacity to recognize a wide variety of antigens and for the surveillance of malignancies. However, cells in the thymus are fragile and sensitive to changes in the external environment and acute insults such as infections, chemo- and radiation-therapy, resulting in thymic injury and degeneration. Though the thymus has the capacity to self-regenerate, it is often insufficient to reconstitute an intact thymic function. Thymic dysfunction leads to an increased risk of opportunistic infections, tumor relapse, autoimmunity, and adverse clinical outcome. Thus, exploiting the mechanism of thymic regeneration would provide new therapeutic options for these settings. This review summarizes the thymus’s development, factors causing thymic injury, and the strategies for improving thymus regeneration.

## Introduction

The thymus is the primary lymphoid organ for T lymphocyte development and maturation that mediates immune defense against foreign antigens, immune tolerance to self-antigens, and immune surveillance on tumor cells (1). The thymic stromal cells include the thymic epithelial cells (TECs), dendritic cells (DCs), macrophages, fibroblasts, vascular endothelial cells (ECs), and connective tissue cells that form an extracellular matrix (2). The network-like structure formed by thymic stromal cells regulates the development, differentiation, maturation, migration of thymocytes, and functional T-cells (3, 4).

Both cTEC and mTEC are morphologically and functionally distinct and mediate different aspects of T cell development. For instance, cTECs are required to commit early thymocyte precursors to the T cell lineage and induce a positive selection of diverse and functionally distinct T cells by unique antigen-processing systems and thymus-specific proteasome subunit (5). While mTECs regulate the migration of positively selected thymocytes from the cortex into the medulla *via* chemokines CCL19 and CCL21 and are also crucial for establishing T cell tolerance *via* ectopic expression of peripheral tissue-restricted antigens and cooperation with dendritic cells (6). Thus, the interactions between the cTEC, mTEC, and developing thymocytes are necessary to ensure a functional, self-tolerant T cell repertoire.

The thymus, as a highly complex structure, is constantly undergoing acute stresses and regeneration cycles. Regardless of its regenerative capacity, the thymus undergoes aged-related involution, a process that includes reductions in thymic mass, loss of thymic structure, and disorganization to thymic architecture (7). The thymus is also very sensitive to insults, including infections, stress, and cytoreductive therapies. Thymus dysfunction results in T-cell mediated cellular immunity defects predisposing to infections and autoimmune diseases (8, 9). Studies showed that insults to the thymus negatively affect TCR repertoire, diminish peripheral T cell pool with subsequent detrimental consequences on immunity and immunotherapies, implicating that rejuvenating an injured thymus to recover immunocompetence is crucial not only for efficient immune responses against pathogens and tumor antigens but also for optimal responses to immunotherapies (10). Several new strategies to improve thymus regeneration were proposed with the finding of mechanisms governing thymus repair. Thus, an improved understanding of thymus structure, development, and involution can enhance our knowledge of current and prospective therapies on thymus regeneration. This review will highlight reported research data on thymic degeneration and the recent advances in thymic rejuvenation.

## Thymus Development

The thymus is a bi-lobed organ located in the thorax, comprising of two similarly sized lobes. Each lobe of the thymus is subdivided into lobules, each containing an outer cortex and an inner medulla (2, 4, 11). The thymus and parathyroid originate from third pharyngeal pouches, with their development involving a series of epithelial/mesenchymal inductive interactions between neural crest-derived mesenchyme and endoderm. During the 6th week of gestation, the endodermal lining of the ventral wing of the third pharyngeal pouch forms a pronounced sacculation that subsequently detaches from the pharyngeal wall, giving rise to the thymic primordia. The thymic primordia, along with the lower parathyroid glands, migrate in a caudal and medial direction as development continues from the 6th to the 9th week (1, 2, 4). After the migration is completed, the thymic endodermal-derived epithelial cells develop into stellate elements, forming a sparsely fibrous reticular meshwork. The surrounding mesenchymal elements form a capsule around it and later form a trabecular, dividing the organ into lobules. By the 10th week, small lymphoid cells originating from the fetal liver and the bone marrow populate the thymus, forming a cortex and a medulla. Concurrently, small tubular structures called medullary duct epithelium develops and later matures into Hassall’s corpuscles (1, 11, 12).

Notably, the formation of cTEC and mTEC is crucial for thymus development, providing a home for recruited lymphoid progenitor cells (11, 12). Recent studies in birds and mice have shown that cTEC and mTEC originate from common endoderm dual-potent progenitor cells (13–15). Bleul et al. also proved that cTEC and mTEC could be induced from a common progenitor cell in the thymus after birth (12). Various transcription factors, including FoxN1, Tbx1, Pax1, Pax3, Pax9, Hoxa3, Eya1, and Six1, regulate the TEC development and thymus organogenesis; the most critical is fork-head box protein-N1 (FOXN1) which has been shown to modulate TEC patterning in the fetal stage and TEC homeostasis in the post-natal thymus (16). Foxn1 regulates the transcription of various target genes essential for thymic function, including Notch ligands (17). FoxN1 deficiency disrupts the thymic architecture and thymic T cell development (18). Interestingly, administration of recombinant FOXN1 protein (rFOXN1) into HSCT recipient mice increased the number of TECs, resulting in enhanced thymopoiesis and increased number of functional T cells in the periphery (19).

It is also documented that abnormalities of other transcription factors such as Pax1 or Pax9, Hoxa3, and Tbx1also lead to impaired thymus organogenesis (20), implicating the essential role of these factors in thymus development. Further studies are therefore necessary to elucidate tissue specific expression of these genes, their roles in TECs development and how they can be employed to boost thymic function.

Several issues on thymopoiesis and the transcriptional factors that regulate T cell development and repertoire remain to be further explored. Fortunately, recent advances in the field of thymus biology using new single-cell transcriptomic and epigenomic technologies have enhanced our understanding of mouse and human T cell development. Notably, postnatal thymopoiesis is dependent on the steady migration of bone marrow-derived hematopoietic progenitors to the thymic parenchyma *via* the blood circulation and their cell surface adhesion molecules (21). The thymic microenvironment gradually drives these multipotent progenitor cells to the T cell lineage and induces proliferation to increase the pool of T cell precursors. A recent study examining immature postnatal thymocyte populations in humans using single-cell RNA sequencing (scRNA-seq) provided insight into the heterogeneity of early T cell precursors and their transcriptional dynamics. Their studies identified two non-proliferative populations that are present in the thymus; TSP1 and TSP2. Of these, the TSP1 population (Lin^-^CD34^+^ CD44^hi^CD7^-^CD10^+^) transcriptionally corresponds to murine TSPs based on the expression of chemokine receptors (CCR7 and CCR9) and transcription factors (HOXA9, MEIS1, and MEF2C) and is postulated to represent the canonical T cell precursors that differentiate into early T cell precursors (ETPs) when they encounter Notch activating signals within the thymic microenvironment. In contrast, the TSP2 population expressed the Notch target genes CD7 and CD3E before thymic entry, contributes to the T lineage differentiation process (22).As thymocytes progress from the multipotent ETP stage, they lose non-T cell lineage potential and commit to the T cell fate. A recent study by Shin et al. showed that Runt domain-related (Runx1 and Runx3); are essential for early T cell development in mice from uncommitted to committed stages, mostly activating T-lineage and repressing multipotent progenitor genes. Runx1 and Runx3 are coexpressed in the thymic T progenitor cells, bind to highly overlapping genomic sites, and have redundant, collaborative functions regulating genes pivotal for T cell development (23).

### cTEC

The thymic cortex provides a microenvironment that supports the generation and T cell antigen receptor (TCR)-mediated selection of CD4+, CD8+, and TCRαβ+ thymocytes. cTEC expresses chemokine ligands such as CCL25, CXCL12 and secretes cytokines such as IL-7, essential for the early development and intrathymic positioning of thymocytes (24–26). Notch signal is also implicated in mediating the interaction between TECs and thymocytes, where Notch1 activation in T cell progenitors is initiated through interaction with Notch1 ligands Delta-like 4 (DL4) to activate signaling pathways, leading to the proliferation and migration of thymocytes (27). Lymphoid progenitor cells entering the thymus cortex express neither the TCR complex nor the CD4 or CD8 markers, a stage termed double negative (DN).Maturation progresses with the acquisition of CD4 and CD8 markers, generating the CD4+, CD8+ double positive (DP) cells. This complex process involves several stages before DN1 (CD44+, CD25-), DN2 (CD44+, CD25+), DN3 (CD44-, CD25+), DN4 (CD44-, CD25-) proliferate and differentiate into DP thymocytes (28).At the thymus’s cortical-medullary junction, DP thymocytes recognize antigen peptide-MHC-I/II molecular complexes and differentiate into CD4+ or CD8+ single-positive (SP) thymocytes (29, 30).

cTECs expressed proteins such as the lysosomal protease Prss16 and Cathepsin L has been demonstrated to be essential for generating an immunocompetent repertoire of CD4+CD8− T cells (31). Others have also posited that cTEC’s positive selection process of CD8+ T cells is linked to its expression of thymoproteasome β5-thymus (β5t) subunit (31). It is worth noting that even though β5t expression was confined predominantly within the cTEC^hi^ (32), β5t+cTECs at the cortico-medullary junction of 1-week-old mice was identified to serve as an efficient progenitor for the mTEC lineage, indicating that progenitors resident efficiently enables expansion of medulla in the thymic cortex. Yet, once the medulla has reached its normal cellularity in the postnatal thymus, the differentiation potential of β5t+ precursors to the mTEC lineage is markedly restricted (33).Of note, studies using β5t-deficient mice showed a significant decline in the number of CD8+ SP thymocytes in the thymic architecture with subsequent altered immune responses, suggesting that the thymoproteasome is essential for the production of self-antigens involved in the positive selection of functional CD4−CD8+ T cells (34).Another transcription factor, ThPOK (encoded by Zbtb7b), has recently been identified to promote CD4+ *versus* CD8+ lineage divergence and is implicated to be necessary for determining CD4+ helper fate on TCR-signaled thymocytes and conservation of CD4+ and regulatory T (Treg) cells and agonist-selected lineage gene programs (35). Albeit it has been quite challenging in the past years to investigate the heterogeneity of the TEC population, recent studies using mass spectrometry proteomics and single-cell RNA sequencing established the fact that cTEC expresses Cathepsin L, TSPP, and β5t, while mTEC expresses Cathepsin S, CD40, and Aire (5, 36, 37).

### mTEC

Interaction with mTEC renders self-tolerance of T-cell as indicated by deletion of SP thymocytes showing high avidity with tissue restriction antigen (TRA) expressed on mTEC. In addition to mediating negative selection, mTEC is also involved in Treg cell differentiation. This process is dependent in part on the autoimmune regulatory gene (AIRE) and Fez family zinc finger protein 2 (Fezf2) (38, 39). Accumulated records indicate that the postnatal mTECs contain two major subpopulations that are defined according to their levels of cell surface MHC-II and CD80 molecules: MHC-II_low_CD80_low_ (mTEC_low_) cells and MHC-II^hi^CD80^hi^ (mTEC^hi^) cells (40). AIRE+ mTEC^hi^ subsets are further subdivided into osteoprotegerin-positive (OPG+) and negative (OPG−) subpopulations (41, 42). OPG regulates the cellularity of mTECs and the size of the medullary region in the thymus by attenuating the proliferation of mTECs. Lymphotoxin-like β receptor (LTβR), the receptor activator of NF-κB (RANK) and CD40, are also indispensable for the development and maturation of AIRE+mTEC (42, 43), *via* interaction with relevant ligands on the surface of thymocytes (44).

AIRE promiscuous gene expression of TRAs by mTEC is required to delete self-reactive thymocytes (39). AIRE’s significant role in T cell tolerance is evident from the autoimmune manifestations in AIRE-deficient mice (45). Accumulating evidence from studies has also demonstrated AIRE and Fezf2 co-expression in mTEC^hi^ cells, suggesting their similar mechanistic function to permit the expression of TRAs in the thymus to ensure immune tolerance (39, 46, 47). However, Hiroyuki Takaba et al. in a study showed that Fezf2 directly regulates various TRA genes in mTECs independently of AIRE, as mice lacking Fezf2 in mTECs displayed severe autoimmune symptoms, including the production of autoantibodies and inflammatory cell infiltration targeted to peripheral organs, interestingly, these responses varied from those spotted in AIRE-deficient mice (48).

The exact mechanism of the interaction between mTECs and thymocytes and their subsequent regulatory impact on the fate of TCRs, either toward clonal deletion or Treg cell specification, is still ambiguous and requires further investigation. It is depicted in studies that thymic dendritic cells (DCs), mainly conventional DC (cDC), subsets signal regulatory protein a (SIRPa+) and CD8a+ readily acquired MHC class I and II from TECs to delete self-antigen–specific thymocytes and drive the development of Foxp3-Tregs to mediate negative selection. The study also pointed out that inhibiting PI3K signaling pathway reduced MHC acquisition by thymic CD8a+cDC and plasmacytoid DC but not SIRPa+ cDC, signifying that multiple parameters influence the mechanisms that drive intercellular MHC transfer by thymic DC subsets (49). Likewise, a new subset of medullary SIRPα+ cDCs that express CD301b (SIRPα+CD301b+ cDC2s) was reported by Elise Breed et al. to have potent transcriptional signatures for MHC class II antigen processing. Interestingly, this population depended on signaling *via* the IL-4Rα, and their ablation resulted in decreased clonal deletion, implicating the impact of cytokines from innate-like T cells on self-tolerance (50).

Moreover, a recent study by Tom Sidwell et al. on the developmental lineages of thymic Treg cells and transcription factor BAH2, revealed that loss of BACH2 enhanced the TCR- and IRF4-dependent selection of CD25+Foxp3–Treg cell precursors but attenuated the generation of CD25–Foxp3+ Treg cell precursors, demonstrating that the two developmental pathways have discrete transcriptional and stimulus requirements (51). Masashi Watanabe and colleagues on the roles of the B7–CD28 costimulatory axis on thymic APCs also reported that self-antigen-specific clonal deletion was not impacted by B7 deficiency in any single APC population, but Treg cell development substantially decreased with the absence of B7 on dendritic cells, highlighting the distinct contributions of APCs to the two fates. Their study also showed that different CD28 intracellular cytoplasmic tail motifs were required for clonal deletion *versus* Treg cell development (52). Indeed, Treg cells, as specialized T cell lineage, have a pivotal function in controlling self-tolerance and inflammatory responses, however, several areas on how mTECs mediate Treg development and its crosstalk with tissue-specific genes expressed on mTEC need further investigation (53). A concentrated effort is also required to clarify the factors and mechanisms that regulate thymic DC subsets to acquire MHC and stimulate thymocytes in negative selection.

## Thymic Injury: Factors and Agents

**Figure 1:**
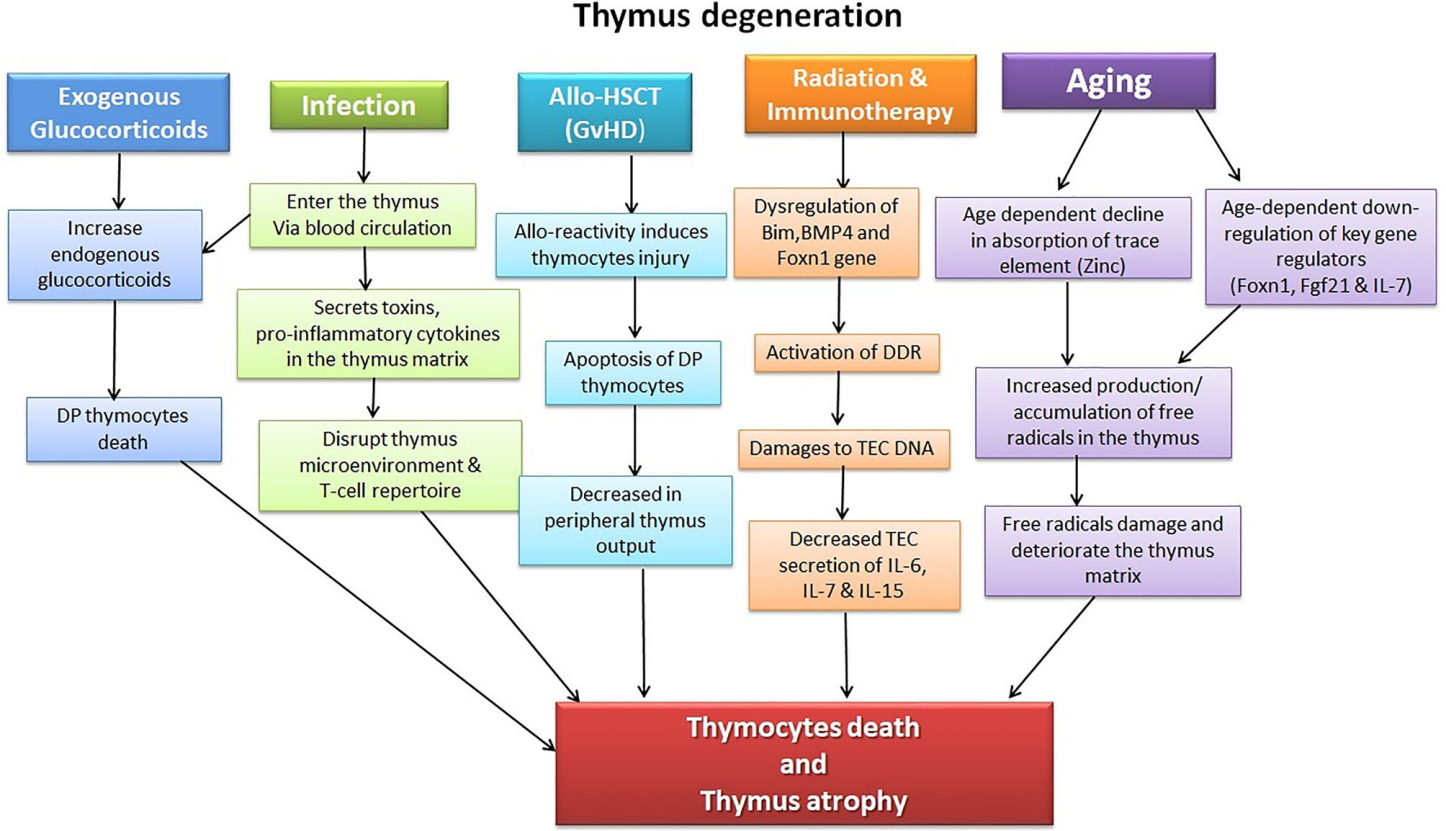
Illustration of factors causing thymic injury. The thymus is a delicate organ sensitive to insults, including chemotherapeutic drugs, corticosteroids, radiation, and pathogens. Allo-HSCT treatment regime and its complications (GVHD) likewise have detrimental effects on the thymocytes. Age-dependent thymic atrophy naturally occurs due to a decline in the thymus’s self-renewal capacity and subsequent accumulation of toxic substances, including free radicals.

The thymus is extremely sensitive to various factors and agents, including acute insults, such as stress, acute infection, glucocorticoids, cytoreductive therapies, or chronic damage, such as chronic infection and age-related thymic involution (**Figure 1**). These factors have a diverse effect on the thymus; for instance, while acute thymic involution results primarily from the loss of cortical thymocytes; chronic atrophy, such as that induced by age-related thymic decline, leads to loss of Foxn1+TECs and thymic atrophy, resulting in the functional deterioration of the TEC compartment (54, 55).

### Acute Injury

#### Glucocorticoids

Glucocorticoid (GCs) is widely used in clinical settings. However, elevated levels of endogenous glucocorticoids under pathological conditions such as stress, infection, Cushing syndrome, and even GCs secreted by TECs induce apoptosis and cause acute degeneration of the thymus (56). Synthetic GCs have previously been shown to influence immune functions by promoting the apoptosis of immature CD4+CD8+ DP thymocytes (57, 58). A recent study also showed that synthetic GCs, including dexamethasone (DEX), damage thymic cellularity and architecture, subsequently leading to thymocyte apoptosis and progressive diminution in DP T cells (59). They mediate these biological effects by binding to intracellular glucocorticoid receptors (GRs) that act through mitochondrial apoptotic pathway activation in DP thymocytes (60). Whereas the CD4+CD8+ DP thymocytes are very sensitive to GCs, as elevation of GCs could almost annihilate the DP population by apoptosis and inhibition of their proliferation, Treg cells are resistant to *in vivo* GCs-induced apoptosis (61, 62).

It was also shown that *in vitro* DEX treatment induced upregulation of most apoptosis-related molecules (caspase-3 and 8) in thymocytes (63). However, pro-and anti-apoptotic molecules expression was increased in Tregs compared to CD4+ T cells (61). The feasible explanation for relative apoptosis resistance of Treg cells to GCs is likely due to the upregulated Bcl-2 expression and basal cytosolic Ca2^+^ level in Tregs, as these were downregulated in DX-induced apoptosis sensitivity CD4+ T cells. Other studies have indicated that adolescent sex hormones such as dihydrotestosterone (DHT) and testosterone also act on the thymus through nuclear receptors and may directly induce thymocytes apoptosis (60). Since GCs have been implicated to play a significant role in the pathogenesis of aged related-thymic involution and thymus injury caused by infection, stress, and malnutrition (64, 65), future studies should be directed to investigate the molecular mechanism underlying the diverse thymocytes response to GC-induced apoptosis, to help develop a new therapeutic strategy to boost thymic function in these settings.

#### Infection

The thymus as an immune organ plays a significant role in the immune response during acute infection; however, the thymus is also a target of multiple pathogens. These pathogens usually enter the thymus through blood circulation, disrupting the thymic structure and altering the T-cell repertoire. Studies showed that acute viral infections, including influenza virus and Epstein-Barr virus, not only affect the thymus microenvironments and cause thymic atrophy but also interference with thymocyte development and increased apoptosis of thymocyte subsequently leading to acute thymic injury (66, 67). Acute bacterial infections, including streptococcus infection can also cause thymic involution by triggering apoptosis in developing thymocytes, while Mycobacterium tuberculosis infection leads to thymic atrophy (68). Other infectious agents, including viruses, protozoa, and fungi, can invade the thymic microenvironment, disrupting thymocytes and peripheral T lymphocytes output to generate central immunological tolerance of the infectious agent-derived antigens (64). Most foreign pathogens destroy the thymic matrix, release inflammatory mediators such as tumor necrosis factor-α, interferon-γ, and induce apoptosis of DP thymocytes (69).Toxins, pro-inflammatory mediators, and soluble factors such as glucocorticoids secreted by these pathogens also lead to thymic atrophy and affect thymic immune response (54). It is worth mentioning that chronic systemic infections such as HIV and cytomegalovirus (CMV) have long-term detrimental effects on thymic structure and functions, leading to thymic atrophy, reduced thymic output, and disruption of the thymic microenvironment and subsequently leading to chronic thymic insult. This mechanism of evading host immune clearance requires further study to identify a better therapeutic way to restore the damaged thymus (66).

#### Allogeneic Hematopoietic Stem Cell Transplantation

Application of immunosuppressive therapy, such as chemo and radio-therapy in cancer management or prior to transplantation, damages not only tumor cells but also has catastrophic effects on healthy hematopoietic cells, peripheral immune cells, and thymic microenvironment resulting in reduced T cell development and repertoire (70, 71). Chemotherapy profoundly impairs thymus function by causing thymus atrophy, reducing T lymphocytes, shrinking the thymic lobules, and decreasing the production of naïve T cells (72, 73). In clinical settings, a substantial decline in CD31^+^ recent thymic emigrants (RTE) counts and single-joint T-cell receptor excision circles (sjTREC) levels in the peripheral blood were found after chemotherapy. Specifically, both CD31^+^ RTE counts and sjTREC levels decreased to the nadir at the end of chemotherapy and recovered within one year of follow-up (74). Similarly, the combination of total body irradiation with cyclophosphamide chemotherapy damaged the thymocytes and distorted T cell repertoire (75).Certain immunomodulatory medications such as rabbit anti-thymocyte globulin (rATG) also decrease TECs expression and secretion of interleukins with increased thymocytes apoptosis and delayed immune regeneration (76).

Although thymic epithelial cells possess the anti-radiation ability, studies have shown that under hypoxic conditions, the expression of pro-apoptotic factor Bim is usually up-regulated, mTECs apoptosis increases, radiation resistance is impaired, and cTEC becomes less effective (77). In the early stage of radiation injury, endothelial cells (EC) secrete bone morphogenetic protein 4 (BMP4), which acts on the corresponding receptor BMPR2 on the surface of cTEC and promotes the expression of the FOXN1 gene (78). Conversely, certain chemotherapeutic drugs reduce these factors’ production and further damage the thymus (77, 78). Our recent study demonstrated that JAK inhibitor blocked thymus regeneration after irradiation-induced injury. This effect, dependent on the JAK-STAT pathway, inhibited growth factors and their receptors, subsequently suppressed the proliferation of TECs (79).

Additionally, it is reported graft-*versus*-host disease (GVHD) has a tremendous damaging insult on the thymus. The known postulated mechanism of thymus GVHD is that allot-reactive T lymphocytes recognize the allogeneic tissue antigen and attack the recipient’s thymus to mediate tissue injury (80), which subsequently leads to DN thymocytes differentiation inhibition with an increase in apoptosis of DP thymocytes (81). Preclinical studies have shown that GVHD impaired the size and function of mature Aire^+^mTEC^hi^, impacting the normal expression of TRAs, thus negatively affecting the maintenance of central tolerance (81). Data from early studies (82, 83) indicate that GVHD-associated thymic damage decreases thymic cellularity and loss of thymic negative selection and persistence of self-reactive T cells. In the murine Allo-HSCT model, GVHD induced TECs apoptosis by IFNγ secreted from donor T cells in a STAT -1 and caspase-dependent manner (84).

Yet, in other previous studies (85, 86), a systemic abrogation of IFNγ signaling in transplant recipients exert variable and unpredictable effects on the outcome of GVHD on the thymus, implicating the need for further investigation to underpin the molecular mechanisms by which GVHD exert insult on thymocytes. Interestingly, Dudakov et al. demonstrated that murine-GVHD results in depletion of intrathymic group 3 innate lymphoid cells (ILC3s) necessary for thymic regeneration. Loss of thymic ILC3s resulted in a deficiency of intrathymic IL-22, thereby inhibiting IL-22-mediated protection of TECs and impairing recovery of thymopoiesis (80). Notwithstanding the benefits of chemo and radiation-therapy in Allo-HSCT, these therapies’ detrimental consequences impair the thymic function and inhibit the thymus’s ability to rejuvenate.

### Chronic Injury

#### Aging

Immune system functionality declines significantly with advancing age, increasing susceptibility to infections and other chronic inflammatory disorders. Age-related involution is characterized mainly by progressive regression of thymic size and structure, resulting in impaired thymopoiesis, restricted TCR repertoire, ineffective central tolerance, and accumulation of senescent memory T cells, consequentially leading to innate immune cell-induced chronic inflammation (87–90). Generally, aging also affects various stages of T cell development, from age-related alterations in HSC, disruption in thymic microenvironment niche, changes in key signaling molecules that modulate thymopoiesis, reduction of T cell differentiation activity, to the subsequent decline in T cell output and immune function (91). Firstly, concerning HSC, increasing age is associated with a decreased ratio of lymphoid-to-myeloid cells, resulting in fewer early T-cell progenitors (ETP) entering the thymus from the BM, consequently causing thymic atrophy with decreased thymic output (92). In addition, in aging thymic epithelial cells, the expression of some critical regulatory genes is down-regulated. FOXN1, fibroblast growth factor 21 (fgf21), IL-7, and the growth factors of TECs declined significantly (93). These contribute enormously to age-dependent thymic involution and consequently deteriorate senile immunity.

What is more, the aged thymus declines in both TECs and thymocytes as age-related involution negatively affect thymocyte development and selection, leading to the diminution in DP and SP thymocytes (88, 90). Animal model studies evaluating the impact of age-involution on stromal compartment depicted an atrophied thymus due to substantial loss of TECs, predominantly CD205^+^ cTEC and UEA-1^+^ mTEC, in addition to significant down regulation of various TEC markers such keratin and MHC class II (89, 94). With age, the atrophied thymus also declines in its capacity to establish central tolerance, thereby causing increased self-reactive T cells to escape to the periphery and participate in the process of inflammaging. Moreover, the reduced thymic output and peripheral oligo-clonal expansion of memory T cells in the aging thymus result in an overall contracted TCR repertoire diversity thereby inducing immune insufficiency (immunosenescence) (95).

Several studies reported age-dependent thymic atrophy abates bone marrow hematopoietic stem cells’ function to differentiate into T lymphoid progenitor cells (96, 97). TEC’s self-renewal ability in the thymic matrix of the elderly decreases sharply, whiles the number of fibroblasts and adipocytes increases (98, 99). Due to aerobic metabolites’ accumulation in the aging thymus, TEC trans-differentiates into fibroblasts and adipocytes through epithelial-stromal trans-differentiation. Another explanation is that the intestinal tract’s ability to absorb trace element zinc (Zn) decreases with age, increasing free radicals production (87). Additionally, the thymic stromal cells lack hydrogen peroxide reductase and are sensitive to oxygen free radicals, which may further damage the thymic matrix (100).

#### COVID-19 and the Aged-Thymus

Studies on the global pandemic of coronavirus disease 2019(COVID-19 have reported a higher frequency of severe symptoms and mortality in elderly patients, implicating a direct relationship between age and COVID-19 prognosis (101). Similar observations were also shown in SARS-CoV-1 (102), MERS (103),and experimental models of SARS-CoV-2 infection (104). It is proposed that immunosenescence and inflammaging are high-risk factors for severe COVID-19 in the elderly, suggesting that the age-related clinical severity of COVID-19 is due to impaired antiviral immune function and excessive self-damaging immune reactions in the elderly (101, 105). Others also posited that the advanced age population is highly prone to viral infection because of a lower functional capacity of phagocytes to eliminate pathogens and activate the adaptive immune response (106, 107). Functional impairment of T and B-lymphocytes, coupled with an increase in exhausted T cells, contributes to poor clinical outcomes of COVID-19 (108, 109). This is consistent with studies that reported lymphopenia in patients over the age of 60 with severe COVID-19 (110), although this could also be due to SARS-CoV-2 spike proteins directly interacting with CD26 on T cells, leading to T cell apoptosis and immune dysfunction (111).

One primary physiognomy of the immunosenescence is a low-grade proinflammatory state, with increased levels of IL-6, IL-1, TNF-α, and C-reactive protein (112, 113), which lead to cytokine storm syndrome and multiple organ failure (114). Studies have shown similarities between aging blood cytokine profile and that observed in severely ill COVID-19 patients, which appears to play a vital role in poor COVID-19 prognosis (115). Interestingly, the cytokine storm syndrome in COVID-19 patients is mainly characterized by the IL-1, IL-6, and TNF-α (116, 117), among which the serum TNF-αlevel is negatively correlated with T cell function by downregulating the expression of CD28 (118). Conversely, a study by Cuvelier et al. showed no significant increase in IL-6 and IL-10 but indicated that the lack of thymic reactivation in older SARS−CoV−2 infected patients contributes to a worse prognosis. Their data showed that an increase in thymic mass, partly triggered by enhanced IL-7 levels, is a beneficial adaptation to virus-induced lymphopenia. Unfortunately, this adaptation diminishes in advanced-aged patients, possibly contributing to the higher mortality observed in old individuals (119).

## Regeneration Strategies of the Thymus

The human body requires a robust immune system to fight off infections to ensure survival throughout life. Since the thymus efficient function declines substantially due to aging and other environmental factors, it is critical to elucidate the available therapeutic methods of thymus regeneration. Few clinically proven therapies have been reported to restore the thymus function, while pre-clinical studies postulated other options to rejuvenate it. Currently available regenerative strategies of the thymus involve pathways that either specifically target non-hematopoietic cells such as TECs or modulate bone marrow hematopoietic progenitors to mediate regeneration of the thymus (19, 120). Others have also exploited alternative therapeutic strategies, including hormonal therapies, transplantation of thymic tissue, thymic organoids, and artificial thymus transplantation, to boost thymic function (121, 122). As already discussed, thymic injury is caused by several factors with diverse mechanism resulting in different outcomes. Normally, injury in hematopoietic origin may lead to transit and spontaneous thymus reconstitution, whereas injury in non-hematopoietic origin such as TECs may require both endogenous and exogenous strategies to rejuvenate thymic function (59, 112). This section of the review will briefly highlight both previous and current therapeutic strategies based on their targeted cells (**Table 1**).

**Table 1:** Summary of thymus regeneration therapeutic strategies.

Therapies	Mechanism	Targeted cells	Reference
Sex steroid inhibition(SSI)	Application of sex hormone inhibitors or sex steroid ablation alleviates thymic apoptosis, enhances thymus growth and proliferation of peripheral T-cells.	Thymocytes	(142, 172, 174)
Precursor T-cells	Direct injection of transduced OP9-DLL1 or DLL4 ex vivo generated pre-T cells promotes thymocytes proliferation and maturation in the thymus. T-iPSCs enhance proliferation, differentiation and functionality of antigen-specific T cells.	Thymocytes	(177, 178) (178, 183)
Thymus transplantation	Transplantation of artificial thymic stromal cells; TEPC with intracellular components, reconstructs thymus structure and boost thymic function.	TECs	(184–186)
Keratinocyte growth factor (KGF)	Binds to FGFR2b to activate the PI3K-Akt signaling pathway to induce proliferation and differentiation of TEC.	TECs	(123, 124)
Interleukin 22	Stimulates TEC proliferation and survival *via* radio-resistant RORγ and FOXN1 up-regulation. Accelerates thymus recovery *via* AIRE activation and regulation of JAK/STAT3/Mcl-1 pathway.	TECs	(133, 134, 137)
Growth hormone (GH)	Activates JAK2/Stat1, 3,5 to induce cell proliferation, increases thymic cellularity and promotes thymus regeneration. IRS phosphorylation activates PI3K-Akt and MAPK signaling pathways to enhance the survival of TECs.	TECs and TSC	(141, 146, 172)
RANKL(TNFSF11/TRANCE)	Controls self-tolerance in the mTEC microenvironment by regulating the activation of classical and non-classical NF-κB pathways *via* TRAF6.	mTEC	(147–149)
Epidermal growth factor receptor (EGF)	Activation of MAPK & PI3K-Akt to enhance proliferation and survival of epithelial cells. Regulates the production of TEC-derived cytokines within the thymus.	TECs	(150–154)
Bone morphogenic protein 4 (BMP4)	Promotes TEC proliferation and maturation by inducing the expression of FOXN1 and its downstream target delta-like 4 (DLL4) in cTECs.	TECs	(78)
Interleukin 7 (IL-7)	Modulate mTEC-derived CCR7 ligand expression to boost normal thymocytes development and maturation. Increases progenitor T cells to promote the expansion of naive and memory CD4+ and CD8+ T cells.	TECs	(26, 157)

SSII, sex steroid inhibition; Pre-T, precursor T cells; TEPC, thymic epithelial progenitor cells; ESC, embryonic stem cells; TSC, thymic stromal cells; T-iPSCs, induced pluripotent stem cells derived from antigen-specific T cells; ILC3, Group 3 innate lymphocyte; EGF, epidermal growth factor receptor; IRS, insulin-like receptor substrate; FGFR2b, fibroblast growth factor receptor 2b; RORγ, retinoic acid-related orphan receptor; FOXN1, fork-head box protein-N1; AIRE, autoimmune regulator; TRAF6, tumor necrosis factor receptor.

### Thymic Epithelial Cell Regeneration

TEC performs critical functions in the normal thymus and during rejuvenation after thymic injury. *In vitro* synthesis of essential molecules secreted by thymus, a recombinant humanized chemokine (CCL25, CCL21), IL-22, IL-7, and other cytokines have apparent efficacy in treating damaged thymus. Studies aimed at recovering TEC function *in vivo* with various hormonal or cytokine treatments are already in progress. Moreover, several of these approaches have been tested in phase I or phase I/II clinical trials.

#### Keratinocyte Growth Factor

Fibroblast growth factor 7 (FGF7), also known as a keratinocyte growth factor (KGF), has been reported in studies to promote the proliferation and differentiation of TEC (123). Mesenchymal-derived cells, such as fibroblasts, can secrete KGF to bind to the fibroblast growth factor receptor 2b (FGFR2b) to activate the PI3K-Akt signaling pathway and promote the proliferation of TEC cells (124). Exogenous use of KGF enhanced T-cell lymphopoiesis in Allo-HSCT mice with protective effects on thymic stromal cells. In an experimental Allo-HSCT model, KGF prevented thymic injury and GVHD by maintaining gastrointestinal tract integrity and acting as a “cytokine shield,” which helps prevent subsequent pro-inflammatory cytokine generation (125). Simona W. Rossi et al. also elucidated in their studies the impact of KGF to induce *in vivo* transient expansion of thymic epithelial cells to promote differentiation of TECs. The protective and proliferative effects of KGF on thymocytes could be because KGF signaling in TECs activates both the p53 and NF-kB pathways, resulting in the transcription of several target genes essential for TEC function and T-cell development (126, 127). Pre-clinical data also demonstrated that recombinant human KGF could enhance thymic epithelial tissues’ regenerative capacity and protect them from a wide variety of toxic exposures. These cytoprotective effects of KGF have been attributed to its ability to decrease thymic infiltrated T-cells and strengthen TEC barrier integrity (128).

#### Interleukin-22

Interleukin 22 (IL-22), a cytokine primarily associated with the maintenance of barrier function and induction of innate antimicrobial molecules at mucosal surfaces, is reported in a number of studies to play significant roles in TEC rejuvenation (80, 129, 130). We reported that up-regulation of intra-thymic IL-22 positively correlated with thymus regeneration in mice treated by total body radiation (TBI), an effect triggered by depletion of CD4 and CD8-DP thymocytes (131). Others depicted similar results; in one of the studies, IL-22 knock out (IL-22−/−) mice exposed to sublethal TBI or received Allo-HSCT demonstrated enormously impaired thymic regeneration with noticeably declined thymocytes, TECs, and non-TECs (132, 133). Interestingly, a similar outcome was identified in our recent study on human transplantation. The study analyzed the dynamic change of plasma IL-22 level and assessed recovery of thymic output function by detecting T-cell receptor excision circles (TRECs) level. The findings suggested that the dynamics of plasma IL-22 levels correlate with the recovery of thymus function in human allotransplants (134). Evidence from these studies implicates that cross-talk between IL-22 and TECs is crucial to mediate T-cell immunity’s reconstitution after injury.

**Figure 2:**
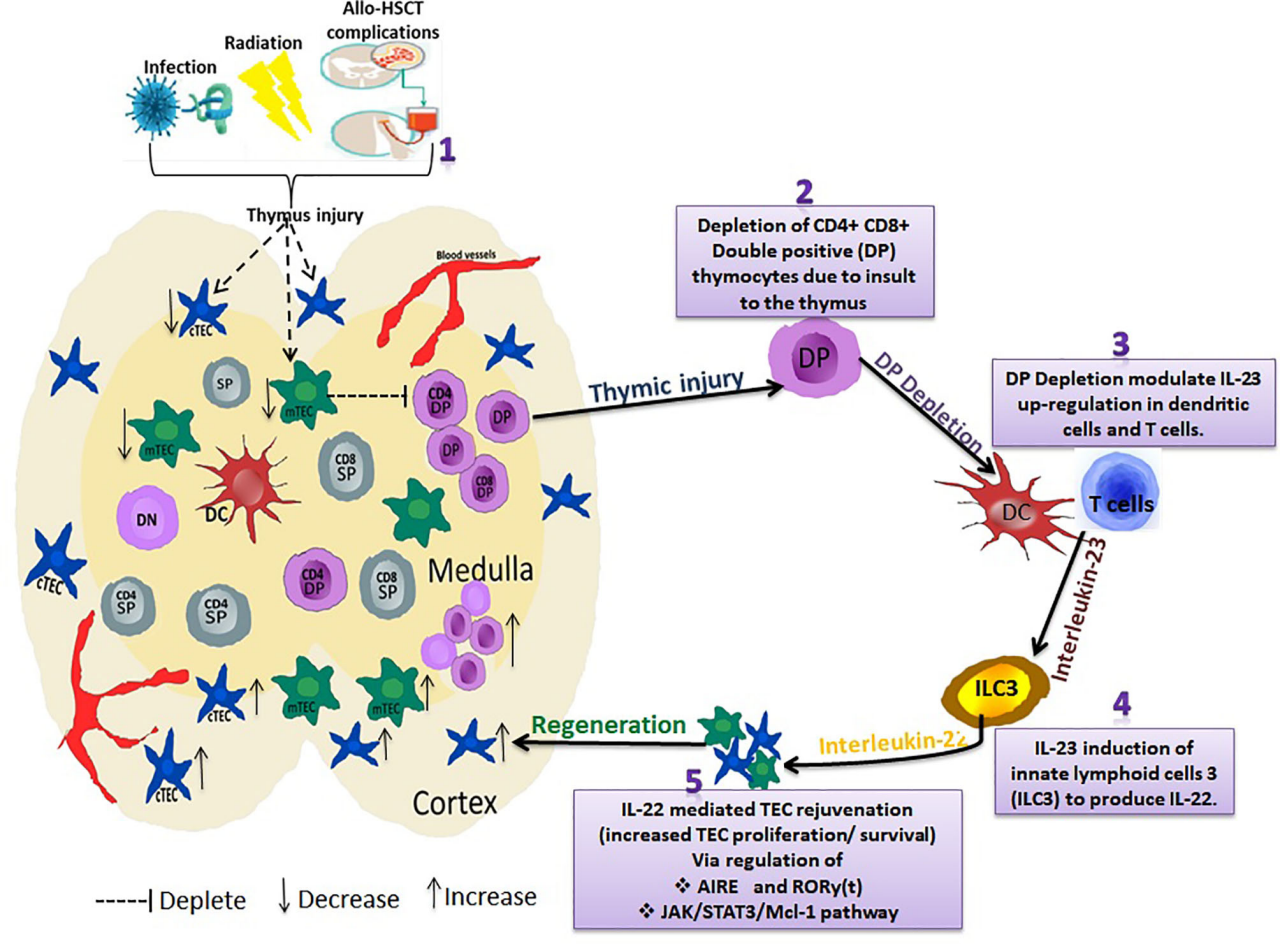
Interleukin-22 promotes endogenous thymic regeneration. Exposing the thymus to infection, irradiation, and Allo-HSCT treatment regime (1) results in depletion of CD4+ CD8+ Double positive (DP) thymocytes (2) DP depletion modulates IL-23 up-regulation in dendritic cells and T cells (3). IL-23 then induces the group 3 innate lymphoid cells (ILC3) to produce IL-22 cytokines (4). IL-22 regulates AIRE and RORy(t) *via* JAK/STAT3/Mcl-1 pathway (5). This subsequently causes a significant increase in TEC proliferation/survival, thus boosting TEC and thymus regeneration.

Compelling evidence indicates that IL-22 modulatory effect on thymocytes promotes TEC’s proliferation and survival *via* the upregulation of radio-resistant RORγ and FOXN1 (16). RORγ (t) is expressed in T cells and is critical for thymocyte development by regulating DP thymocytes survival and genes that control thymocyte migration, proliferation, and T cell receptor selection (135). Dudakov et al. also reported that IL-22 signaled through TECs in an IL-23–dependent manner and promoted their proliferation and survival *via* up-regulating RORγ(t)^+^CCR6^+^NKp46^–^ lymphoid tissue inducer cells (136).We recently reported IL-22 accelerated thymus remodeling after transplantation by regulating the JAK/STAT3/myeloid cell leukemia sequence 1 (Mcl-1) pathway (137). STAT3 signaling is crucial for the survival of mTECs and maintenance of thymopoiesis (138). STAT3 binds the promoter region of Mcl-1, modulating IL-22 to regulate the proliferation of TEC. Moreover, Mcl-1, a member of the Bcl-2 family, is an anti-apoptotic gene-regulating survival of hematopoietic cells. Mcl-1 is also essential for the survival of mature cortical and medullary TECs and the maintenance of thymic architecture. A screen of TEC trophic factors in organ cultures also showed that epidermal growth factor upregulated Mcl-1 *via* MAPK/ERK kinase activity, providing a molecular mechanism for the support of TEC survival (139). Hence, IL-22 protective and proliferative effects on TECs could be IL-22 regulatory impact *via* JAK/STAT3/Mcl-1 path since activation of this pathway inhibit apoptosis on TECs (137, 138, 140). These show that IL-22 might represent a novel strategy in thymic rejuvenation and could restore thymic function (**Figure 2**).

#### Growth Hormones

Growth hormone (GH), often referred to as ‘insulin-like growth factor 1 (IGF-1)’, stimulates the growth of virtually all body tissues, including bone, and it is crucial for protein synthesis and fat metabolism. A study by Tasaki et al. showed that both plasma levels of IGF-1 and the number of cells expressing IGF-1R and FOXN1 in the thymus correlated with age-associated thymic involution. Their studies indicated that the number of cells expressing IGF-1R and FOXN1 in the naive aged thymus was significantly lower than in juvenile animals (141), implying the necessity of extrinsic factors such as IGF and FOXN1 in the preservation of the TECs repertoire and TEC’s reconstitution. Exogenous GH administration in mice increased thymic cellularity, delays thymic involution, and positively regulates T cell migration (142).

Similarly, in a clinical trial study (NCT00071240), human recombinant GH (somatropin) administration in HIV-infected patients increased thymic cellularity and peripheral immune response (143) with a significant increase in both naive CD4+ and CD8+ T cells. A recently published clinical study (TRIIM Trial) of 10 healthy men (51- 65 years) treated with recombinant human growth hormone (rhGH), dehydroepiandrosterone (DHEA), and metformin also showed improved thymic function with protective immunological changes and improved risk indices for many age-related diseases (144). A further study (TRIIM X trial) involving both men and women from 40-80 years of age is currently in progress (NCT04375657) to help elucidate the therapeutic benefits of rhGH. Ghrelin and IGF-1 are postulated to be involved in the GH pathway, where ghrelin promotes the secretion of GH, and IGF-1 is one of the primary mediators of the effects of GH (142). IGF-1 binding to its receptor IGF-1R activates the JAK2/StAT1, 3, 5 signaling pathway to induce thymocyte proliferation (137, 145). Another hypothesized classical pathway besides IGF-1R activation is the phosphorylation of the insulin-like receptor substrate (IRS). Uncoupled IRS activates PI3K-Akt and MAPK signaling pathways and promotes thymic epithelial cells’ survival (146).

#### RANKL

TEC development is a sophisticated and gradual process controlled by the extrinsic and intrinsic signal regulatory network. mTECs express a diverse set of tumor necrosis factor receptors (TNFRs), and three of them, including the receptor activator of NF-κB (RANK), CD40, and LTBR, cooperatively control the thymic medullary microenvironment and self-tolerance (41). Accumulating evidence indicates that the TNFR family members are essential in determining mTEC formation and development. Besides, exogenous recombinant RANKL enhances the thymus’ recovery after acute injury in mice (44). In the embryonic thymus, RANKL signals provided by CD4+CD3−lymphoid tissue inducer (LTi) cells promote CD80−AIRE−mTECs developing into CD80+ AIRE+ mTECs. Disruption of the RANKL-RANK signaling in the postnatal thymus reduces mature UEA-1+CD80^hi^ MHCII^hi^ mTECs (43). RANK activates NF-κB pathways *via* TNFR-associated factor 6 (TRAF6). TRAF6-deficient mice showed severe destruction of medullary architecture and loss of Ulexeuropaeus agglutinin1 (UEA-1) +mTECs. TRAF6 also activates TGF-β activating kinase 1 (TAK1), which activates the IKK complex. These mechanistic pathways are posited to regulate RANKL-RANK mediation in mTEC (147, 148). Notably, a study revealed that transplantation of RANKL−/− thymus to immune-deficient mice caused severe inflammatory cell infiltration and abundant production of autoimmune antibodies (149), meaning that the abnormality of RANKL-RANK signaling may result in mTEC development anomalies and T cells self-tolerance failure. Thus, targeting the RANKL-RANK signaling pathway in the thymus’ medullary microenvironment could lead to the discovery of new therapy to boost thymus reconstitution.

#### Epidermal Growth Factor

In the normal human thymus, the epidermal growth factor receptor (EGF-R) is expressed by sub-capsular, cortical, and medullary epithelial cells (150). Application of exogenous recombinant EGF promotes the proliferation of TECs and thymus repair after acute injury in mice. EGF binds to EGF-R, a protein tyrosine kinase (PTK). It initiates a series of events that include activation of PTK activity of EGF-R, phosphorylation of the EGF-R, and phospholipase C-3’ (123). EGF also activates the classical MAPK and PI3K-Akt signaling pathways, promoting epithelial cells’ proliferation and survival (151). It is worth remembering that transforming growth factor-a (TGF-a) is structurally and functionally related to EGF, and both are known to complaisantly modulate cytokines secretions in human TECs (152). Pre-clinical studies showed that TGF-a is localized in the mTECs and thymic Hassall’s bodies, while EGF-R is localized to TECs throughout the thymus (153, 154). Their data highlighted that TGF-a and EGF are critical regulatory molecules for producing TEC-derived cytokines within the thymus and may function as critical modulators of human T cell development *in vivo*. Of note, studies demonstrate the possible impact of EGF on preserving TECs and ensuring thymic reconstructions (129).

#### BMP4 and IL-7

In addition to the thymus rejuvenation strategies discussed, bone morphogenic protein 4 (BMP4) and interleukin-7 (IL-7) are also targeted as therapeutic regulators of thymic regeneration since they are implicated in several studies to play a significant role in TEC proliferation and preservation. Briefly, BMP4 is expressed in fibroblasts, ECs, and cells in the thymus (155). BMP4-induces the expression of FOXN1 and its downstream target delta-like 4 (DLL4) in cTEC. Both FOXN1 and DLL4 are essential for the development of TECs and thymocytes (78). This means, regulating BMP4 therapeutically could enhance proliferation and maturation of TEC to mediate thymus regeneration *via* upregulation of FOXN1 and DLL4. Likewise, IL-7, mostly found on TECs, is critical for normal thymocytes’ development and maturation. Studies indicate that mice deficient in Il-7 had a substantial decline in various subsets of T lymphocytes. IL-7 administration seems to increase progenitor T cells and naïve T cells, subsequently augmenting thymus repair (130, 156). Alternatively, it was revealed that IL-7 receptor (IL-7r) also play an essential role in the generation of microenvironments required for thymic DCs and T-cell development, as Il7r-/- mice exhibited a significant decrease in mTEC-derived CCR7 ligand expression and severe defects in thymic corticomedullary structure and mTEC development (157).

Some clinical trials (NCT00684008, NCT00477321, NCT01190111, and NCT01241643) have revealed that administration of a glycosylated recombinant human IL-7 (rhIL) enhanced T cell recovery in either HIV or HCT patients *via* acceleration of thymocytes and T cell subsets reconstitution (158–162). Precisely, a phase I/IIa dose-escalation study (NCT00477321) reported that rhIL-7 application in HIV-1-infected patients was safe, well-tolerated, and transiently promoted the expansion of naive and memory CD4+ and CD8+ T cells (159). A similar study (NCT00839436) in patients with idiopathic CD4+ lymphopenia also showed that rhIL-7 could increase the number of circulating CD4+ and CD8+ T cells (163). Besides, a study (NCT00684008) in T cell-depleted allogeneic HCT patients treated with rhIL-7 depicted a substantial increase in peripheral CD4+ and CD8+ T cells implicating immune-regenerative properties of rhIL-7 (164). Though evidence from studies reveals possible therapeutic benefits of BMP4 and IL-7, additional studies are required to clarify this approach of thymus-dependent therapy.

### Thymocytes Recovery

#### Sex Hormone Inhibitors

Increased sex steroid and hormone levels act through their nuclear receptors, causing thymic involution. Their effect manifests during puberty, during which the rate of thymic involution upsurges rapidly (142, 165).Primarily, androgen receptors (AR) and estrogen receptors are expressed in the hematopoietic and stromal compartments of the thymus (166).Although their exact effect and mechanism on thymic involution are still unsettled, evidence from studies demonstrated that sex steroids such as testosterone could induce apoptosis of CD4+CD8+ DP thymocytes *via* the upregulation of TNF-α (167); while estrogens (estradiol) induce thymic atrophy by eliminating early thymic progenitors (Flt3^+^Sca-1^+^c-Kit^+^ population in the bone marrow) and inhibiting the proliferation of beta-selected thymocytes (168). These claims are supported by studies in animal models that showed that sex steroid inhibition (SSI) increases thymic cellularity, restores thymic architecture and organization, and enhances thymopoiesis (169, 170). Castration has also been shown to increase FoxN1 protein levels and Foxn1^+^Ly51^-^CD80^+^TECs, restore the level of CD4+CD8+SP, and immature CD25^+^CD44^+^CD117^+^ thymic progenitors, subsequently enhancing thymic rejuvenation (171).

Recent experimental studies have also proved that the use of SSI or surgical destruction of the hypothalamic-pituitary-gonadal axis can promote thymus growth and increase peripheral T cells’ diversity (172). Similar observations were reported in clinical trials where sex steroid ablation (SSA) in prostate cancer patients enhances thymic function with a significant rise in naïve CD4+and CD8+T cells, NK cells, and TRECs (173). Also, a pilot study reported that luteinizing hormone-releasing hormone agonist (LHRH-A) goserelin considerably increased neutrophil and lymphocyte numbers within the first month of post-transplantation and subsequently promoted T cell repertoire regeneration and peripheral T cell function without exacerbating GVHD (174). Even though these studies’ evidence seems promising, further studies are required to apply sex steroids inhibitors to alleviate thymic involution without causing hormonal deficiency disorders.

#### Precursor T cells and Thymus Transplantation

Direct injection of precursor T cells (pre-T) *in vitro* is proven to accelerate patients’ immune reconstruction, especially using the OP9-DL1 system for generating precursor T cells ex vivo (175, 176). Previous work has shown that pre-T cells can be generated using ex vivo co-culture of hematopoietic stem cells (HSCs) with ectopically transduced OP9-DLL1 and DLL4, two critical factors for thymocytes proliferation (177, 178). Previous studies on the generation of OP9-DL1–derived T-cell precursors from umbilical cord blood (UCB) or HSCs, revealed that two distinct progenitor subsets;CD34^+^CD45RA^+^CD7^++^CD5^-^CD1a^-^(proT1) and CD34^+^CD45RA^+^CD7^++^CD5^+^CD1a^-^ (proT2), were able to home to, settle, and differentiate in the thymus of recipient immunodeficient mice (179). Murine Allo-HSCT recipients of OP9-DL1–derived T-cell precursors showed increased thymic cellularity and substantially improved donor T-cell pool. OP9-DL1–derived T-cell precursors gave rise to host-tolerant CD4+ and CD8+ populations with normal T-cell antigen receptor repertoires, cytokine secretion, and T-cell reconstitution after transplantation (180). Although previous methods of the adoptive transfer of T-cell precursors appeared to restore the T-cell–mediated immunity after HSCT, highly expanded T cells have not yet proven to be therapeutically effective in clinical settings, mainly due to losses of function (exhausted T cells) and antigen specificity (TCR destabilization) to nonspecific T cells that occur during the ex vivo manipulation of patient T cells (181).

Several attempts have been made to overcome these impediments by reprogramming antigen-specific T cells to generate iPSCs (T-iPSCs) (182). Nishimura and coworkers demonstrated that T-iPSCs and the subsequent redifferentiation to mature functional CD8+ T cells are not just possible but could also serve as highly proliferative naive cells with elongated telomeres and exert T cell functions which can rejuvenate mature antigen-specific T cells. Immunological assays data from their study also showed that redifferentiated CD8+ T cells exerted T cell functions such as cytolytic activity, IFNγ secretion, and degranulation in a normal manner when stimulated with their specific antigens (183).Though the study focused on CD8+ T cell rejuvenation, exploring this concept on CD4+ helper or regulatory T cells in future studies can provide new avenues to enhance adoptive T cell immunotherapy.

This is also attainable by targeting the molecular signaling pathways that control T-cell development from HSPCs, and those in the thymic microenvironment that integrates multiple niche molecules to ensure T cell diversity and repertoire. A typical example is the synergistic interactions between Notch ligand Delta-like 4 and vascular cell adhesion molecule 1 (VCAM-1), which boost Notch signaling and progenitor T-cell differentiation rates. Shukla et al. showed recently that an engineered thymus-like niche using the above-mentioned thymic signaling factors enables *in vitro* production of mouse Sca-1+cKit+ and human CD34+ HSPC-derived CD7+ progenitor T-cells capable of *in vivo* thymus colonization and maturation into cytokine-producing CD3+ T-cells (178). This engineered thymic-like niche may offer an opportunity for *in vitro* analysis of human T-cell development as well as clinical-scale cell production for future development of immunotherapeutic applications.

Additionally, thymus transplantation and *in vitro* bioengineering can either be utilized to boost the recovery of thymic function or treatment of patients with congenital thymic atrophy (184). The most commonly used method is the transplantation of artificial thymic stromal cells, mainly thymic epithelial progenitor cells (TEPC), containing intercellular interaction components to support T cell development (185). Studies of animal models have demonstrated that, *via* an *in vitro* reprogramming technique, TEPC can induce human embryonic stem cells to form TEC and then transplant them into mice under the regulation of FOXN1, IL-7 (130, 156), BMP4 (78), FGF and EGF to form thymus structure (155, 185). Another method is to remove all the thymus cells and leave only the matrix components, which can be recombined with artificial thymic stromal cells and T lymphoid progenitor cells to form functional thymus (186).

It is encouraging to know that significant advances in T-iPSCs bioengineering technologies (182),including genome-edited master iPSC lines (187), 3D thymic culture generated antigen-specific anti-tumor T cells iPSCs (122), iPSC-derived NK cells (188, 189), CAR-T engineered T- IPSCs (190), TCR and HLA reprogrammed T-iPSCs (191, 192) and inactivation of recombination activating gene 2 (RAG2) in the T-iPSCs (187), have broadened the clinical applicability of adoptive cell immunotherapy and facilitated the development of “iPSC-derived off-the-shelf tumor-specific T cells” cellular therapeutics for the management of several malignancies. Others have also demonstrated the use of Foxn1-induced TECs (iTECs), particularly Foxn1-reprogrammed embryonic fibroblasts (FREFs) to generate a functionally competent thymus organ that robustly supports T-cell development and repertoire (193, 194). It was initially shown in transgenic mice that enforced Foxn1 expression is sufficient to convert primary mouse embryonic fibroblasts (MEFs) into various subsets of TECs that expresses the relevant markers required to promote full T-cell development (193). Injection and engraftment of iTECs with two types of promoter-driven (Rosa26CreERT and FoxN1Cre) Cre-mediated FREFs into the thymus of an old mice was recently shown to rejuvenate the aged thymus by increasing thymopoiesis, along with reduced senescent T cells and autoreactive T cell-mediated inflammation (194). Though these immune rejuvenation therapeutic strategies indubitably have setbacks, they appear promising with potential goals to contribute to thymus regeneration.

## Concluding Remarks and Future Perspectives

The thymus’ regenerative capacity declines substantially in acute and chronic injury, resulting in long-term immune deficiency and infection susceptibility. Although progress has been made in immune reconstruction after acute thymic injury, there are still some limitations and challenges; several mechanistic pathways on thymic rejuvenation are still not well elucidated experimentally. For instance, regulatory T cells’ effect in the thymus medulla on central tolerance formation is still ambiguous, and how mTEC mediates Treg development is not fully understood. Also, the regulatory mechanism and the influence of tissue-specific genes expressed on mTEC require further study. Nevertheless, with the current rapid advancement in scientific research, there are high aspirations that better regenerative strategies would be discovered to promote endogenous recovery of the thymus. A balanced regenerative approach promoting positive and negative selection will be crucial to enhance the thymic function since a biased regenerative strategy may singly improve positive or negative selection. The subsequent research’s primary mission should be addressed to further develop and advance the methodology for regenerating thymic function in patients subjected to thymus injury, thymic degenerative ailments, and thymectomy.

## Author Contributions

KX and BP contributed to the concept, analyzed data, and revised the manuscript. MD and LL did literature retrieval, collected data, and wrote the manuscript. JS and QL did literature retrieval. All authors contributed to the article and approved the submitted version.

## Funding

This study is supported by the National Natural Science Foundation of China (81871263 to KX, 81970159 to BP) and The Natural Science Foundation of the Jiangsu Higher Education Institutions China (20KJA320002 to BP).

## Conflict of Interest

The authors declare that the research was conducted in the absence of any commercial or financial relationships that could be construed as a potential conflict of interest.

## Publisher’s Note

All claims expressed in this article are solely those of the authors and do not necessarily represent those of their affiliated organizations, or those of the publisher, the editors and the reviewers. Any product that may be evaluated in this article, or claim that may be made by its manufacturer, is not guaranteed or endorsed by the publisher.

## Abbreviations

TECs, thymic epithelial cells; CCL, chemokine ligands; Allo-HCT, Allogeneic hematopoietic cell transplantation; KGF, keratinocyte growth factor; RANK, receptor activator of NF-κB; TEPC, thymic epithelial progenitor cells; TSC, thymic stromal cells; EGF, epidermal growth factor receptor; FGFR, fibroblast growth factor receptor; FOXN1, fork-head box protein-N1; AIRE, autoimmune regulator.
